# Pre-operative ambulatory measurement of asymmetric lower limb loading during walking in total hip arthroplasty patients

**DOI:** 10.1186/1743-0003-10-41

**Published:** 2013-04-20

**Authors:** Alicia Martínez-Ramírez, Dirk Weenk, Pablo Lecumberri, Nico Verdonschot, Dean Pakvis, Peter H Veltink

**Affiliations:** 1 Mathematics Department, Public University of Navarra, Pamplona, Spain; 2 Institute for Biomedical Technology and Technical Medicine (MIRA), University of Twente, P.O. Box 217, Enschede 7500 AE, The Netherlands; 3 Department of Orthopaedics 800, Radboud University, Nijmegen Medical Centre, Postbox 9101, Nijmegen 6500 HB, Netherlands; 4 Department of Orthopedic Surgery, Medisch Spectrum Twente, PO Box 50000, Enschede 7500 KA, The Netherlands

**Keywords:** Instrumented force shoes, Gait parameters, Hip osteoarthritis, Total hip replacement, Vertical ground reaction forces

## Abstract

**Background:**

Total hip arthroplasty is a successful surgical procedure to treat hip osteoarthritis. Clinicians use different questionnaires to assess the patient’s pain and functional capacity. Furthermore, they assess the quality of gait in a very global way.

This clinical evaluation usually shows significant improvement after total hip arthroplasty, however, does not provide objective, quantifiable information about the movement patterns underlying the functional capacity, which can currently only be obtained in a gait laboratory. Instrumented force shoes can quantify gait velocity, ground reaction forces and the gait pattern easily in an outpatient setting.

The main goal of this study was to investigate how mobility characteristics during walking, relate to gait velocity and questionnaire outcomes of patients with hip osteoarthritis in an outpatient setting.

**Methods:**

22 patients with primary osteoarthritis of the hip selected for a total hip arthroplasty participated in this study. For each patient the Harris Hip Score, the Traditional Western Ontario and the McMaster Universities osteoarthritis index were administered. Subsequently, the patients were instructed to walk through the corridor while wearing instrumented shoes. The gait velocity estimated with the instrumented force shoes was validated measuring the time required to walk a distance of 10 m using a stopwatch and a measuring tape as a reference system. A regression analysis between spatial, temporal, ground reaction force parameters, including asymmetry, and the gait velocity and the questionnaires outcomes was performed.

**Results:**

The velocity estimated with the instrumented shoes did not differ significantly from the velocity measured independently. Although gait parameters correlated significantly with velocity, symmetry index parameters were not correlated with velocity. These symmetry index parameters show significant inter-limb asymmetry during walking. No correlation was found between any of the variables studied and questionnaires outcomes.

**Conclusion:**

Inter-limb asymmetry can be evaluated with the instrumented shoes supplying important additional information about the individual gait pattern, which is not represented by gait velocity and questionnaires usually used. Therefore, this new ambulatory measurement system is able to provide complementary information to gait velocity and questionnaires outcomes to assess the functional capacity of patients with hip osteoarthritis.

## Background

In the field of orthopedics, osteoarthritis (OA) of the hip is one of the most common and frequent diseases which produces greater restrictions on mobility performance and walking ability in patients over age of 55 years. It is estimated that 1 in 10 of the population who are 60 years or older have significant clinical problems that can be attributed to osteoarthritis. For both males and females the incidence of osteoarthritis rises steeply after the age of 50 peaking in the 70–79 age group. For similar age groups and using radiographic diagnosis the prevalence of osteoarthritis hip was 9.90% in the Netherlands [1]. It has been shown that walking ability is positively related to the way in which patients develop a proper role in everyday life [2,3]. Total hip arthroplasty (THA) is a successful surgical procedure to relieve hip OA. THA usually results in a significant and relevant improvement in functional capacity of patients [4,5].

Currently, clinicians use several standardized and validated questionnaires filled out by the patient, such as the Harris Hip Score (HHS) and Traditional Western Ontario and McMaster Universities osteoarthritis index (WOMAC) [6,7] to evaluate mobility performance, activities of daily living, pain, as well as the satisfaction and welfare of the patient in order to compare the pre and the post-operation situation [8-11].

This evaluation is not based on objective physical measurements but depends on the subjective opinion of the patient, the physiotherapist or clinician, so it is difficult to perform an accurate and objective assessment [12]. Moreover, the evaluation with questionnaires does not provide information about the motor control performance underlying the functional capacity.

Gait analysis is a useful method for assessing functional deficits before and after THA [13,14]. Measuring the left-to-right difference in vertical ground reaction force, the weight loading asymmetry during walking can be quantified and related to the status of impairment [15,16].

During walking, the lower extremity joints are cyclically loaded. Anomalous joint motion can develop abnormal loading, which has been connected to osteoarthritis disease. The development of OA has a large impact on joint loading [17]. It is important to study the mechanical loading of the lower limb joints to understanding the development and progression of OA. Ground reaction forces provide indirect information about internal joint loading because peak loads on hip joint during gait coincide with peak ground reaction forces [16]. Ground reaction forces have been used to quantify atypical limb loading for individuals before and after hip arthropasty [16-18]. A pattern of limping and asymmetries during gait due to weakness and pain has been shown in THA patients at a pre-operative stage [18-20]. Patients are sometimes reluctant in loading their affected limb during the weight acceptance phase of walking [21,22]. This may cause an additional stress on the unaffected lower limb and may accelerate the development of osteoarthritis in the unaffected lower limb [19]. Previous studies [14,23,24] have focused in postoperative functional outcome with no preoperative data. However, it is important to know how patients walk before surgery in order to tailor rehabilitation programs after surgery for potential recovery of normal walking patterns [15,25].

Consequently, there is a clinical need for objective physical measurements to evaluate the force distribution on both lower limbs and the functional progress in patients who will undergo a THA.

Objective functional mobility analysis can currently only be performed in a specialized and dedicated gait laboratory, using force platforms and optical systems [13,14,26]. These expensive laboratory systems are not generally available for clinical assessment in orthopedic practice. In addition, these systems are not ideal because the number of consecutive steps that can be measured and the freedom to walk is limited due to the length of the force platform. The optical systems used also show restrictions. The line of sight can be easily blocked and lead to problems with visibility [27]. Furthermore, gait mats are relatively new systems that provide spatial and temporal gait parameters. Although these systems are relatively low cost and portable, the number of consecutive strides that can be measured, as well as the temporal and the spatial resolution are limited. Moreover, it can only measure spatial and temporal gait parameters but no ground reaction forces and gait patterns.

A new ambulatory system to measure functionality of patients, without these restrictions, opens new perspectives to evaluate gait parameters in patients with OA before a THA and also to evaluate the functional progress after THA.

Schepers, et al. and van den Noort JC et al. have demonstrated that instrumented force shoes (IFS) are suitable for the measurement of ground reaction forces and foot positions and orientations during walking [27-30].

A meta-analysis of Vissers et al., 2011 has shown that gait velocity and outcome of the HHS and WOMAC questionnaires demonstrate significant changes when comparing pre- to postoperative conditions [6].

The aim of this study is to investigate how mobility characteristics during walking, relate to gait velocity and questionnaire outcomes (HHS, WOMAC) for patients with hip osteoarthritis in an outpatient setting. For this purpose, we evaluated the use of the IFS for quantitative assessment during gait of patients who will undergo a THA. We hypothesized that the IFS parameters and symmetry indices will provide complementary information to the assessment of gait velocity only and the questionnaires outcomes. Moreover, we validated the gait velocity estimate of the IFS using an independent measurement.

## Methods

### Subjects

Twenty two patients with hip OA participated in this study (ten females and twelve males, age: 63 ±10 years (mean ± standard deviation), body mass 84.3 ±11.2 kg and height 1.63 ± 0.34 m)

Patients with osteoarthritis that had been selected to undergo a primary total hip arthroplasty were recruited from Medisch Spectrum Twente (Enschede, the Netherlands).

The inclusion criteria were age between 50 and 80 years, primary unilateral osteoarthritis of the hip and a total hip arthroplasty planned within the next 4 months.

The exclusion criteria were a contra-lateral THA, any kind of leg arthroplasties, rheumatoid arthritis, any neurological disorder, other degenerative diseases, revision/re-operations of primary hip prosthesis planned or the inability to understand instructions or the questionnaire.

The study protocol was approved by the Medical Ethics Committee (METC) of the Medisch Spectrum Twente, (Enschede, The Netherlands) and full written consent was obtained from all participants.

### Data collection procedures

The measurement sessions were performed in the department of Orthopaedic Surgery at the Medisch Spectrum Twente. Subjects were instructed to walk repeatedly at their preferred speed through a corridor between a predefined start and end point, 10 m. apart at a constant speed while wearing the IFS. The gait velocity estimated with the IFS was validated measuring the time required to walk the distance of 10 m using a stopwatch and a measuring tape as a reference system. In order to control the initial relative positions of the feet, the subject was asked to position the feet against a line on the floor before each walking trial. Three successful trials were collected per subject. The subject had to start 2.5 meters before the start mark and walk 2.5 meters past the finish mark. The stopwatch was started as soon as the subject’s foot crossed the start line and recording was stopped when the person’s second foot crossed the finish line. In this way, the average gait velocity for all trials was calculated independently from the IFS from distance walked and walking time (gait velocity (GV) = distance / time).

**Figure 1:**
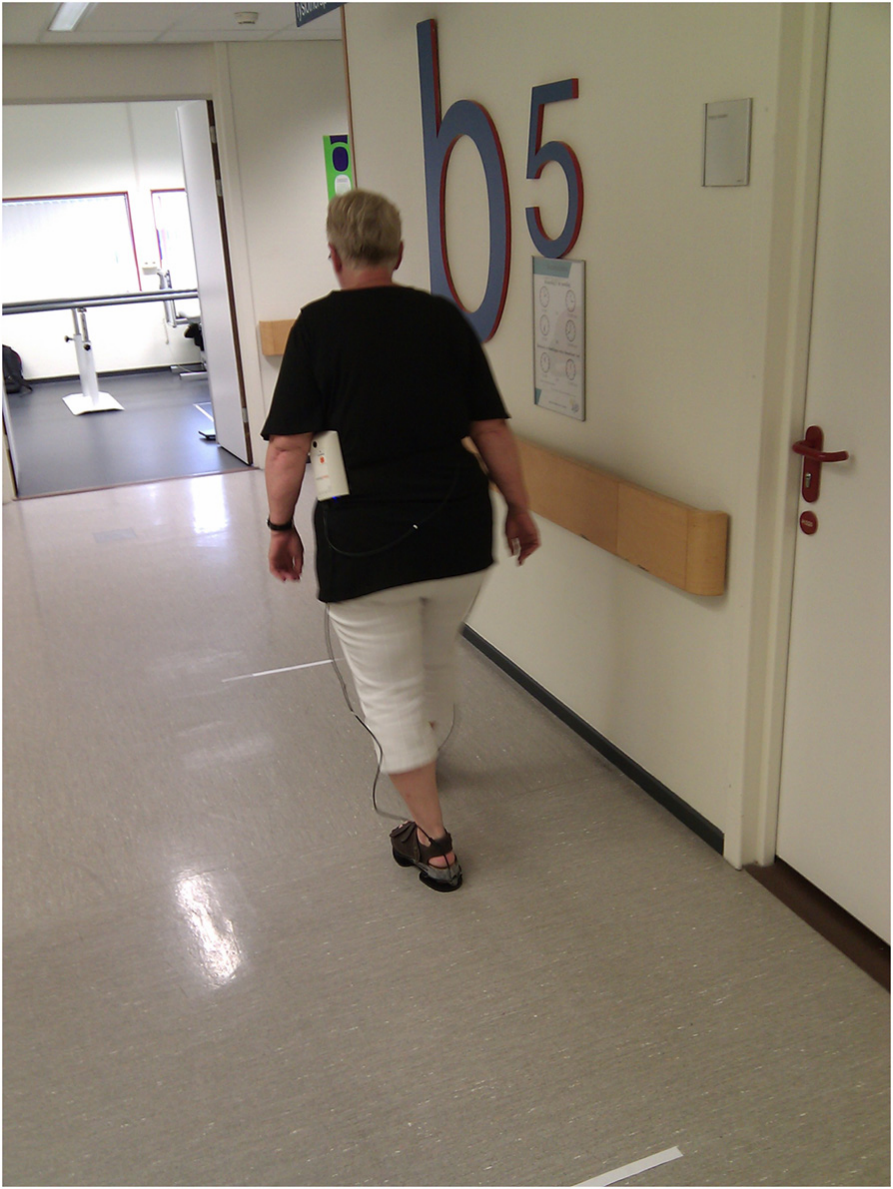
**Measurement Setup.** Hip OA patient during a measurement session performing a walking test.

Figure 1 shows a patient during a measurement session performing a walking test.

### Questionnaires

Subjects were asked, with the researcher’s supervision, to complete 2 questionnaires that are validated to evaluate hip function in THA patients: the Dutch version of the HHS [9], and the WOMAC [6,7]

### Instruments used

The ambulatory measurement system used in this study consisted of an IFS (Xsens Technologies B.V., Enschede, the Netherlands) for 3D measurement of forces and torques under the foot, as well as 3D kinematics of the foot. The complete measurement system is built in a shoe sole allowing complete freedom of movement. The measured data is sent wirelessly to a PC or laptop, via an on-body hub (Xbus master)

The IFS is adjustable for shoe size and it measures the ground reaction force (GRF). The inertial and magnetic measurement systems (IMMS) on the shoes are used to track foot movements with a sample frequency of 50 Hz. These IFS have been validated and used before successfully in a gait study in stroke patients [31], and patients with knee osteoarthritis [30]. It has been demonstrated that the IFS provide reliable accurate measurements of 3D-ground reaction force, position and orientation during gait in healthy subjects [27,32,33]. Moreover, the influence of IFS on the walking pattern of patients with knee OA was investigated by Van den Noort JC et al. [29]. They concluded that the decrease of 8% (0.08 m/s) in walking speed due to wearing the IFS could be regarded as below clinical relevance [29]. Trying to reproduce this result, but taking into account that it had been previously studied and in order not to excessively disturb the patients, the walking speed was measured only in four patients while they were wearing their own shoes.

### Data analysis

All IFS parameters were calculated using MATLAB customized software. Among all possible IFS parameters, the following parameters were selected:

**Gait velocity**: It is computed as the product of stride length and stride frequency measured with IFS during walking at comfortable speed. Stride length was defined as the distance between heel positions of the same foot in a direction parallel to the average walking path at two consecutive instants. Position and orientation can be estimated using inertial sensors by integration of the accelerometer and gyroscope signals [34]. During walking, several initial and final conditions can be assumed to reduce the drift due to the integration of angular velocity to orientation and double integration of acceleration to position [27,28].

**Vertical ground reaction force parameters:** They include early stance maximum (ESM), midstance minimum (MSM), average vertical Ground Reaction Force (vGRF) normalized to body mass (N/kg).

**Time Parameters:** Double support time (DST), stance time (ST) and midstance time (MST).

The parameters were calculated for both involved and uninvolved lower limbs. For instance, DST of the involved lower limb starts with initial contact of that lower limb and finishes with preswing of the uninvolved lower limb.

**Symmetry indices (involved/uninvolved) (SI):** The symmetry index was calculated using the formula:

(1) $$\mathrm{SI}=\frac{\left(V_{I}-V_{U}\right)}{V_{U}}*100\%$$

Where V_U_ and V_I_ are any of the aforementioned parameters for the uninvolved and involved lower limb respectively. Perfect symmetry results in SI = 0 (V_U_, = V_I_), larger positive and negative deviations would indicate a greater asymmetry towards the involved or uninvolved limb.

### Statistical analysis

Descriptive statistics of velocity and gait parameters, mean and standard deviation were calculated. Confidence intervals of symmetry parameters were calculated for the mean and the distribution of the data was summarized and presented in boxplot format. One sample t-tests were calculated to assess whether the symmetry parameters mean is equal to zero with a significance level of 0.05.

Simple linear regression analysis using Pearson’s correlation coefficients (r) was performed taking the IFS spatial, temporal and symmetry parameters, as dependent variables, and the gait velocity (assessed independently from the IFS) and the questionnaires outcomes as independent values. Statistical significance was determined as a P-value of less than 0.05.

To compare the regression slopes of involved and uninvolved limbs and see if they are statistically different we used an ANCOVA test with a significance level of 0.05. In order to test this hypotheses we used a statistical model which describes relationships between the IFS parameter as response variable *Y* (involved and non-involved) and the velocity as an explanatory variable *x* for two groups, indexed by the indicator variable *z* (z = 1 if it is the involved limb and z = 0 if it is the uninvolved limb). The following model will do just that:

(2) $$Y_{i}=\beta_{0}+\beta_{1}x_{i}+\beta_{2}z_{i}+\beta_{3}x_{i}\times z_{i}+\varepsilon_{i}$$

## Results

Measurements were performed on a total of 44 legs. The mean and standard deviation of the parameters for each patient were determined using the data of the three trials.

Mean and standard deviation (SD) of gait cycle parameters and gait velocity measured with the IFS and the reference system are shown in Table 1.

**Table 1:** Gait characteristics of subjects with hip osteoarthritis (N = 22)

**Measure**	**Mean**	**SD**
**Gait velocity independent from IFS (m/s)**	0.87	0.02
**Gait velocity IFS (m/s)**	0.88	0.02
**Gait cycle (s)**	1.27	0.19
**Stride length (cm)**	110.00	23.72
**Stride frequency (stride/s)**	0.80	0.11
**Step length involved (cm)**	55.74	2.04
**Step length uninvolved (cm)**	54.54	1.84

### Questionaires outcomes

Table 2 illustrates the mean, standard deviation (SD) and confidence intervals for WOMAC and HHS questionaires outcomes.

**Table 2:** WOMAC and HHS outcome measures in subjects with hip osteoarthritis (N = 22)

**Measure**		**Mean**	**SD**
**WOMAC**			
	Pain, 0-20	10.7	4.0
	Stiffness, 0-8	4.9	1.9
	Physical function, 0-68	36.3	12.2
	Total, 0-96	51.9	16.9
**HHS**	0-100	51.8	15.4

### IFS parameters

**Figure 2:**
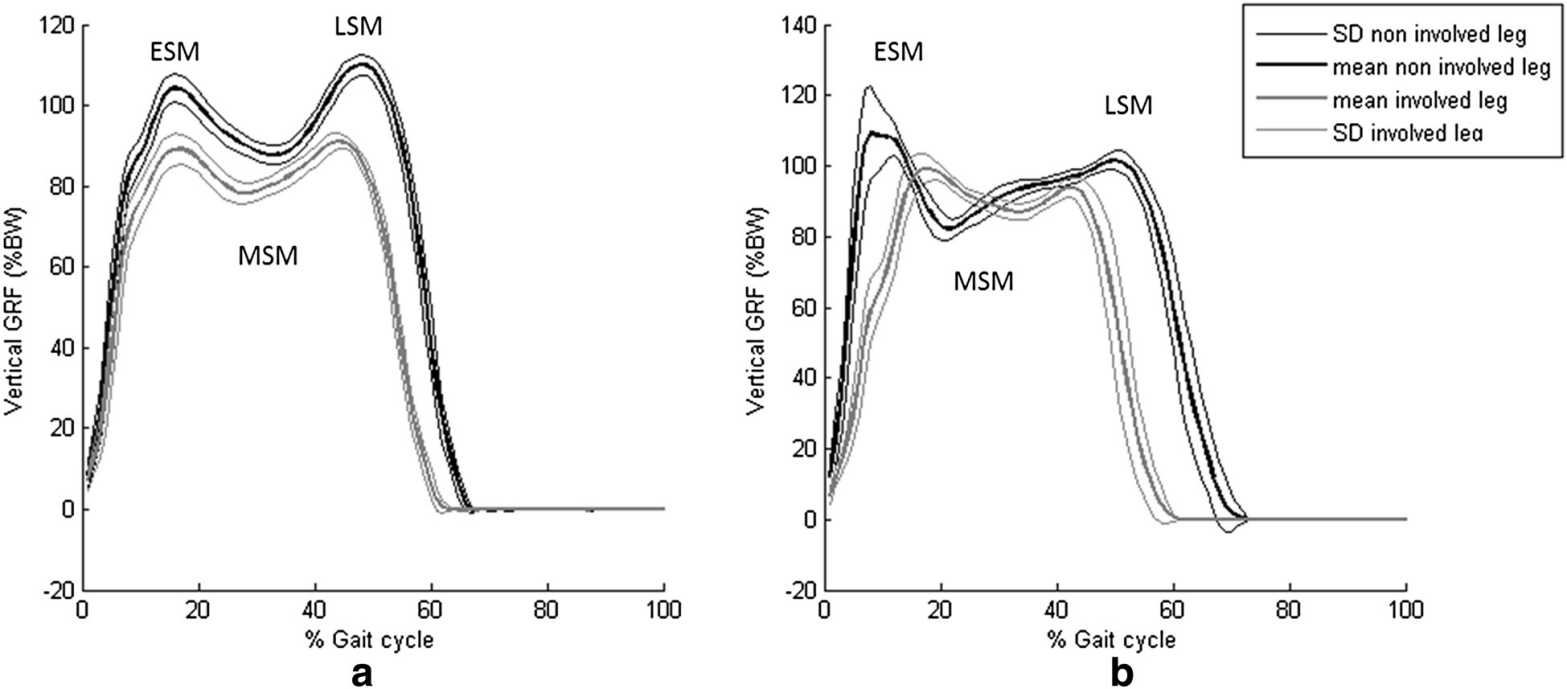
**Vertical Ground Reaction force.** Vertical Ground reaction force: mean and standard deviation (SD) of the vertical ground reaction force of all trials for two different patients.

Mean and standard deviation of the vertical ground reaction force of two different patients are shown in the Figure 2.

Mean and standard deviation (SD) of all patients for involved and uninvolved lower limbs of IFS parameters are specified in Table 3.

**Table 3:** IFS parameters of subjects with hip osteoarthritis (N = 22)

	**Involved (Mean(SD))**	**Uninvolved (Mean(SD))**
**Early stance maximum vGRF (%BW)**	0,97(0,09)	1,01(0,10)
**Midstance minimum Vgrf (%BW)**		
**Average vGRF (%BW)**		
**Stance time (s)**	12,68(3,45)	0,83(0,08)
**Midstance time (% cycle)**	0,80(0,15)	0,45(0,04)
**Double stance time (% cycle)**	25,98(4,80)	29,44(5,77)

### Symmetry of IFS parameters

The SI (involved/uninvolved) were analyzed using equation 1.

**Figure 3:**
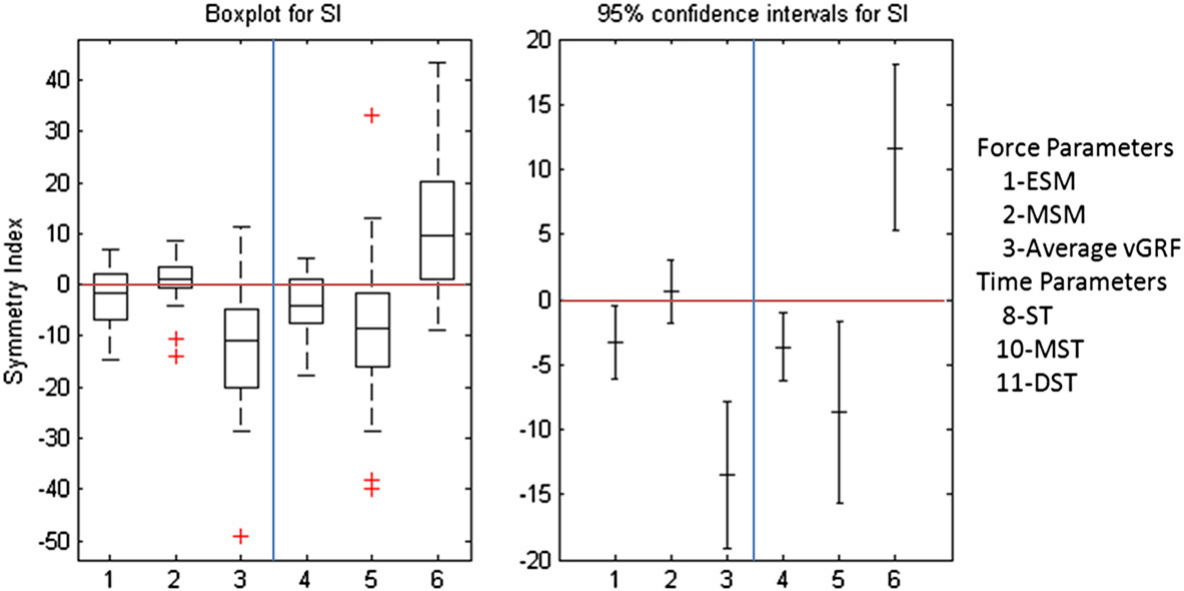
**Boxplot and Confidence intervals.** Boxplot and confidence intervals of symmetry indices of vertical ground reaction force and time parameters for all patients. Perfect symmetry results in SI = 0, larger positive and negative deviations would indicate a greater asymmetry towards the involved or uninvolved limb respectively. The box indicates the lower and upper quartiles with the central line showing the median. The top and bottom lines of the box represent, respectively, the medians for the upper and lower halves of the data and the ‘cat’s whiskers’ represent the highest and lowest values of the distribution, excluding outliers. Outliers are also presented.

Boxplots and confidence intervals of the SI for each gait parameter are plotted in Figure 3.

The negative SI of ESM, ST and MST indicates that the values of these parameters are larger for the uninvolved than for the involved lower limb (p < 0.05). However the SI of DST is positive, indicating that this parameters is largest for the involved lower limb (p < 0.05). MSM did not show asymmetry between both limbs.

The boxplots in Figure 3 show a large variability of symmetry parameters.

### IFS parameters versus GV and questionnaires

#### Gait velocity. Accuracy and precision of the IFS

**Figure 4:**
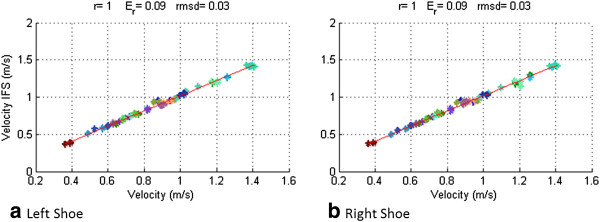
**Velocity.** Velocity measured with the IFS vs GV for all test for all patients for all limbs (left indicated with **a** and right Indicated with **b**). Pearson’s correlation coefficient (r), maximum relative error (Er) and root mean square deviation (rmsd) are indicated at the top of each figure.

Figure 4 shows the velocity measured as the product of stride length and stride frequency measured with the IFS versus GV, assessed independently by measuring the time required to walk the setout trajectory of 10 m using a stopwatch.

The correlation coefficient (r = 0.99) was statistically significant (p ≤ 0.05). This result indicates that there is a strong linear association between the two variables for both right and left shoes. The maximum relative error and the root mean square deviation of velocity estimated from each of both shoes with respect to the reference velocity measurement were 0.09 m/s and 0.02 m/s respectively.

#### IFS parameters versus GV

**Figure 5:**
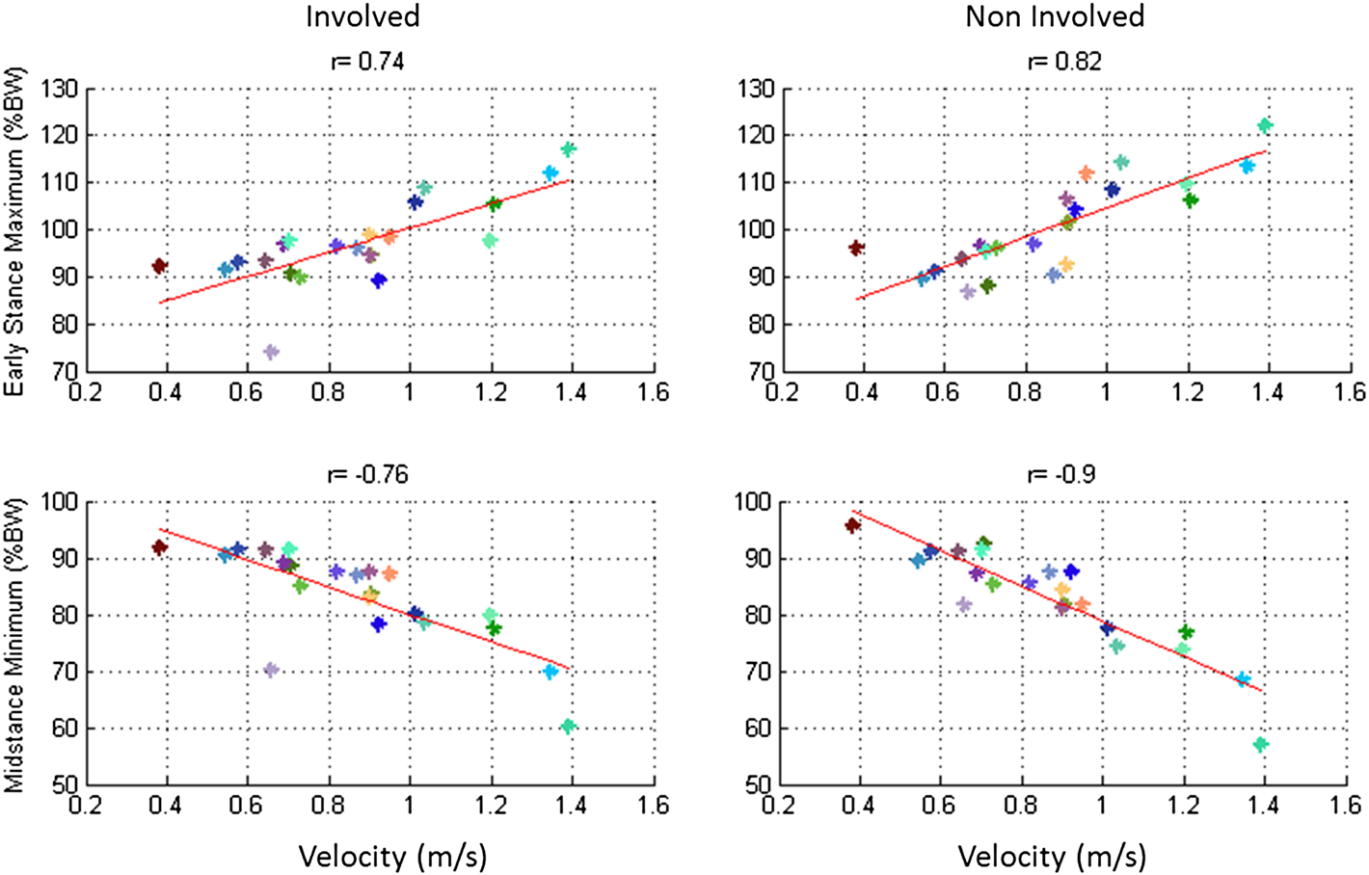
**Linear Regression analysis.** Linear regression analysis of early stance maximum (**a**), midstance minimum (**b**) of involved (left panel) and uninvolved (right panel) limbs of all patients as a function of gait velocity. Each point in the graphic represents the mean of 3 trials developed for each patient.

The ESM had a positive high correlation with GV statistically significant, as we can see in the Figure 5 (r = 0.74 for the involved lower limb and r = 0.82 for the uninvolved lower limb, p ≤ 0.05 in both cases). The MSM (r = −0.76 for the involved lower limb and r = −0.90 for the uninvolved lower limb) had a negative high correlation statistically significant (p ≤ 0.05) for both cases with the GV, as we can see in the Figure 5.

**Figure 6:**
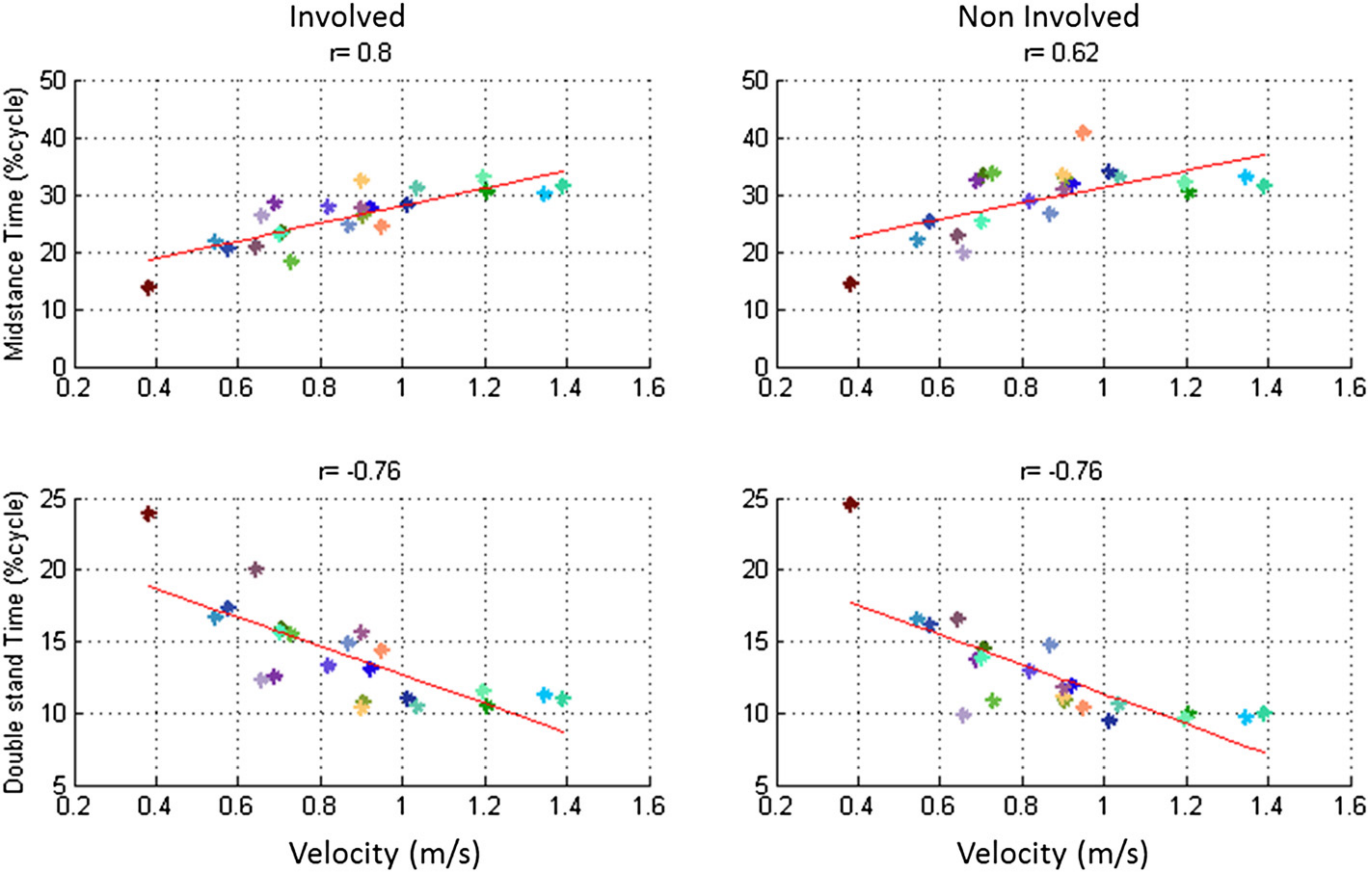
**Linear Regression analysis.** Linear regression analysis of midstance time (**a**) and double stance time (**b**) of involved (left panel) and uninvolved (right panel) limbs of all patients as a function of gait velocity. Each point in the graphic represents the mean of 3 trials developed for each patient.

The MST (%cycle) (r = 0.8 for the involved lower limb and r = 0.62 for the uninvolved lower limb), had a positive high correlation statistically significant (p ≤ 0.05) with GV. The DST (%cycle) (r = −0.76 for the involved lower limb and r = −0.76 for the uninvolved lower limb) had a negative high correlation statistically significant (p ≤ 0.05) with GV, as we can see in the Figure 6.

#### Symmetry versus GV

**Figure 7:**
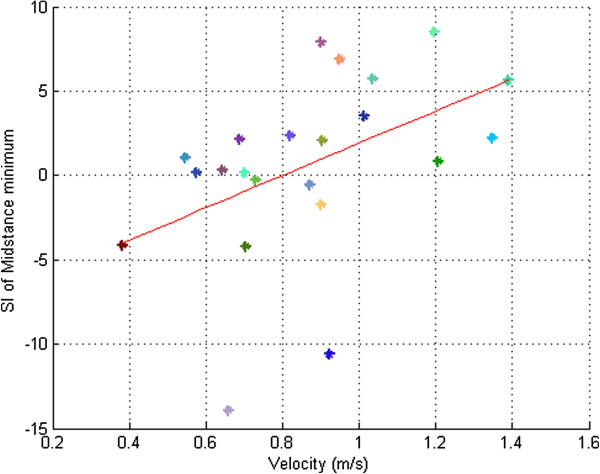
**Linear Regression analysis.** Linear regression analysis of symmetry index of midstance minimum of all patients as a function of gait velocity. Each point in the graphic represents the mean of 3 trials developed for each patient.

SI of the MSM (r = 0.72, p ≤ 0.05) was the only parameter calculated that had a positive high correlation with GV as we can see in Figure 7. No correlation was found between SI of the rest of the parameters measured and GV.

#### IFS parameters versus questionnaires

No correlation was found between any of the variables studied for both involved and uninvolved limbs and the questionnaires outcomes.

## Discussion

This study demonstrates that inter-limb asymmetry provides important additional information about individual gait pattern, which is not represented by gait velocity and questionnaires outcomes.

The velocity estimated with the IFS did not differ significantly from the GV measured with the reference system over the group of patients. It reproduces earlier results of Schepers et al. They demonstrated that IFS gave an accurate estimation of foot positions and orientations during walking [27,28].

Gait velocity is an important determinant of kinematic and kinetic parameters of gait in patients with severe Hip OA [35,36]. ESM, MSM, DST and MST, measured with the IFS correlate significantly with velocity. This correlation was expected for both the involved and uninvolved lower limbs due to the biomechanics of gait at different speeds, which holds for orthopedic patients as well as in healthy subjects [35,37]. Consequently, these parameters do not provide information independent from gait velocity.

It should be noted however that, the SI’s of these parameters were not correlated with velocity, with the only exception being MSM. This indicates that the asymmetry in these parameters cannot be predicted by gait velocity. Therefore, SI provides important additional quantitative information about the functional mobility performance, which is not represented by gait velocity. Moreover, the great variability in the SI of each parameter within our patients indicates that asymmetry differs between patients and is, therefore, important as an independent measure.

The negative SI for ESM, average vGRF, ST and MST indicates greater asymmetry towards the uninvolved lower limb, which means that patients put more weight on the non-affected lower limb throughout the gait cycle. Moreover, a greater asymmetry towards the involved lower limb in the DST is observed. This means that the uninvolved lower limb was loaded for a longer period of time than the involved one. These results are in agreement with those of Talis, Watelain and Hurwitz. The asymmetry of weight bearing might also depend on small changes in the body configuration [15,22]. Joint degeneration is compensated by an increase in pelvis motion and muscle power generation in other lower limb joints. Therefore, the additional stress on the uninvolved lower limb may develop osteoarthritis in that leg. For this reason, it is important to study the mechanical loading of the lower limb joints to understanding the development and progression of OA [19].

The questionnaires usually used reflect different aspects of functionality and the ability of patients to develop activities, but not how to perform them [6]. Patients may try to maintain their functional capacity as normal as possible despite the hardship of pain and discomfort. In the current study we did not find a relation between gait parameters and questionnaires outcomes, which supports the findings of Vissers et al. It is, therefore, important to measure gait parameters in addition to questionnaires to understand how patients walk before surgery in order to compare with their way of walking after the operation and tailor rehabilitation programs for potential recovery of normal walking patterns [15,25].

To verify the IFS accuracy we compared the gait velocity measured with the IFS and independently with the reference system (GV). The velocity estimated with the IFS did not differ significantly from the velocity measured with the stopwatch for each limb, left and right, over the group of patients. This indicates that the IFS do not systematically under- or over-estimate gait velocity.

Previous studies with OA patients have shown that the IFS characteristics are comparable to normal shoes [29,33] in the sense that their influence on the gait pattern is small compared to normal intra-subject variability. Van Den Noort et al. found that the walking velocity of patients with OA of the knees decreased by 8% when walking on the IFS [29]. Trying to reproduce this result, we measured the walking speed in four patients while they were wearing their own shoes. Walking velocity was lower when wearing the IFS (9%), which is comparable to the finding of van den Noort et al. Although the instrumented force shoes are found to be suitable for this investigation, the IFS design needs to be optimized to further reduce the effect on the gait pattern in the clinical setting because of the increment in shoe height and mass and a change in sole stiffness [29]. This could be realized through an exact fit of the instrumented force shoes for all patients, with different shoe sizes and using a more appropriate choice of sole and insoles materials and smaller and lighter force/moment sensors.

Irrespective of the question whether the IFS used in the current study and the study of van den Noort et al. influences walking speed, we conclude that symmetry indices concerning the gait parameters derived from IFS provide additional relevant information about the gait of OA patients that cannot be derived from gait velocity.

Several published investigations agreed that gait mechanics did not return to normal following total hip arthroplasty. Asymmetry of weight bearing could be considered as adaptive behavior; the patients learned not to load their operated lower extremity right after the surgery and continued to do so after recovery [15,24]. In the future, further studies are required to investigate whether the additional gait information found in this study not to be represented by walking speed, is sufficiently sensitive to demonstrate differences before and after THA and whether this information is indeed clinically relevant in the screening before and during rehabilitation after THA.

In this study, we selected to analyze the vertical ground reaction force which is considered to have a greater impact than the other force components [17,18]. In future studies, these ground reaction force components and also the torques under the foot, as well as 3D kinematics of the foot already measured in this study could be analyzed and investigated.

The variation between each test for each of the patients was minimal; the standard deviation was 2.3% of the mean velocity. Therefore, we found it appropriate to analyze the data from all three attempts for each patient. In future studies, it would be interesting to add an analysis of walking at maximum velocity.

## Conclusions

Inter-limb asymmetry can be evaluated in an outpatient setting, supplying important additional information about individual gait pattern, which is not represented by gait velocity and questionnaires usually used. The symmetry parameters calculated in this study are able to provide complementary information to gait velocity and questionnaires outcomes to assess the functional capacity of patients with hip OA. This makes it a new clinical tool useful for tracking the evolution of hip OA patients before and after THA.

## Abbreviations

Total Hip Arthroplasty: THA; Osteoarthritis: OA; Instrumented Force Shoes: IFS; Harris Hip Score: HHS; Traditional Western Ontario and McMaster Universities osteoarthritis index: WOMAC; Medical Ethics Committee: METC; Gait Velocity: GV; Ground Reaction Force: GRF; Inertial and Magnetic Measurement Systems: IMMS; Early Stance Maximum: ESM; Midstance Minimum: MSM; Vertical Ground Reaction Force: vGRF; Double Support Time: DST; Stance Time: ST; Midstance Time: MST; Symmetry Indices: SI; Pearson’s correlation coefficients: r; Standard Deviation: SD; Maximum relative error: Er; Root mean square deviation: Rsmd.

## Competing interests

The author(s) declare that they have no competing interests.

## Authors’ contributions

AM-R: Conception and design, acquisition, analysis and interpretation of data; drafted the article. DW: Acquisition of data. PL: Drafted the article.NV: conception and design. DP: conception and design, acquisition of data. PHV: conception and design, drafted the article. All the authors revised critically the article for important intellectual content and gave the final approval of the version to be published. All authors read and approved the final manuscript.
